# Letter from Paris

**Published:** 1856-04

**Authors:** Robert P. Harris

**Affiliations:** Paris


### LETTER FROM PARIS.

PARIS, Dec. 25th, 1855.

*A few lines for those who contemplate visiting Paris, for improvement in medical knowledge.* By ROBERT P. HARRIS, M. D.

The first requisite for the student or physician visiting Paris to attend its medical school and hospitals, is a knowledge of the French language, particularly that portion of it which is known generally by the term "Medical French." The language should be studied with particular reference to a knowledge of the terms used in medicine and surgery, and of the comparative weights and measures of the French pharmacy. A very imperfect knowledge of the language of every day life will be found quite sufficient for obtaining those comforts that may be required by the stranger upon his first arrival. The main thing is, to be prepared to understand the bed-side talk in the hospitals and the clinical lectures in the amphitheatres. There are but few who come to Paris, excepting those who have been accustomed to read medical books in French, that understand the lectures at first, however perfect their knowledge of the language may be in other respects. The reading of a good medical treatise in French, upon the subject of a lecture, is the cause of much increase in the facility with which the listener understands the language used. For a knowledge of all the terms used, arranged in the vocabulary form, I refer the reader to Nysten's Medical Dictionary, edition of 1855, (Dictionnaire de Médicine par P. H. Nysten.) There are few who come here from America to attend the hospitals, who have not resolved to devote their attention more particularly to one or few of the subjects embraced in the science of medicine. My advice to these is, to read in French some treatise upon their favorite branches, for the purpose of studying the language merely.

The best work that has been written, from which you may gain a knowledge of the Parisian school and hospitals, is that of Dr. F. Campbell Stewart, of Staten Island. The book is somewhat out of date, as it was published years ago, but there is still sufficient to make it of much value to the medical visitor. In it will be found biographical sketches of many of the eminent French physicians and surgeons, some of whom, such as Velpeau, Civiale, Dubois, &c., are still living. To the visitor of the hospitals I recommend the "Guide aux Etudiants en Médicine," a little work by the Secretary of the Faculty of the School of Paris, and one which I have found very little known by the strangers now here. Much time is lost generally by visitors, in their endeavors to find out which hospitals and which cliniques will be the best for them to attend, as the means of information are here very imperfect in this respect, being large hand-bills posted upon the walls of the hospitas, school of medicine and court of the theatre of anatomy.

The portion of Paris in which students generally live, is known as the "Quartier Latin." It is convenient to the Medical School, Dissecting-room, Ecole Pratique, and Hotel Dieu. These are its advantages, as well as its cheapness. In other respects, many other parts of the city are preferable as a residence. By selecting the Rue de l'Odeon, Rue Buonaparte, or some of the widest and cleanest streets of the Quartier, one may live quite comfortably and find his wants well attended to.

The hospitals most visited by students, are Hotel Dieu, Hôpital de la Pitié, Hôpital de la Charité and the Hôpital des Cliniques de la Faculté de Médicine. The Hôpital St. Louis is not much visited during the winter months, but is much frequented in the spring.

In former times, the usual hour for visiting the hospitals, to attend the bed-side cliniques, was 7 in the morning. At present it is at 8, in the most of them, though the time varies with different Physicians, from 7½ o'clock to 9. Eight o'clock is found by the student to be quite early enough at this season, particularly if he is visiting, as I often do, a hospital at nearly three miles distance. As a general rule, but one hospital and one clinique in that hospital can be attended each day. I once attended the bed-side instruction of three physicians and one surgeon in one hospital, on the same morning, and twice I have visited two hospitals on the same day; but this can rarely be done, and is more of a feat than an advantage. All students should pay a visit, if it is only for once, to Hôpital Lariboisiére, a new institution and only completed last year. It is by far the most magnificent establishment of the kind I have ever seen, and I very much doubt if its equal is to be found in any other city.

The physicians and surgeons of Paris, best known in America, are Velpeau, Civiale, Jobert, Ricord, Malgaigne, Dubois, Cruveilhier, Rayer, Cazenave and Andral. The Surgeon whose clinical lectures are the most crowded at the present time, is Nélaton, at the Hôpital des Cliniques. Next to him is Velpeau, at La Charité. Jobert, at Hotel Dieu, and Michon, at La Pitié, have also large classes in attendance. Guersant, the Surgeon of the Hôpital des Enfants' Malades, gives very instructive lectures upon the surgical treatment of maladies in children; he has also quite a large class at his Thursday visit and lecture. Of the physicians, there are a number who are nearly equal, in respect to the size of their audience. Troussseau, at Hotel Dieu, Dubois, (obstetrics,) at Hôpital des Cliniques, and the physicians of La Pitié and La Charité, have all large classes. I have given here the names of those whose cliniques are most frequented by students, and, as a general rule, they are the best lecturers for the student to hear; but there are some hospitals which are very little visited, on account of distance, that have very eminent physicians and surgeons, and whose instructions are perhaps, more valuable, as there is a much better opportunity of studying the cases, on account of their being no crowd of visitors. I particularly recommend, as a surgeon, M. Denouvilliers, the lecturer upon anatomy, one of the surgeons of St. Louis. His service is a very large one, both male and female, and the cases are many of them of great insterest. He is a good operator, and his treatment of his patients has none of that roughness, almost amounting sometimes to brutality, observed in some of the French surgeons. On Wednesday, he examines and treats cases of uterine disease, the patients under his care being mostly from the outside, who go there on that day to be prescribed for.

For those who desire to study the specialities of medicine and surgery, I will state a few facts in point. The principal eye cliniques, are those of Sichel and Désmarres, the latter the most preferable. For uterine diseases, Dr. Gibert, " St. Louis," Mondays; Jobert, " Hotel Dieu," Mondays and Fridays; Denouvilliers, " St. Louis," Wednesdays; Chassaignac, Lariboisiére, Tuesdays and Saturdays. For stone in the bladder and diseases of the urinary organs, Civiale, " Hôpital Necker," on Saturdays. For obstetrics, Dubois, " Hôpital Cliniques de la Faculté de Médicine." For syphilis in its primary form, Ricord, Hôpital du Midi; in its secondary forms, Gibert, at " Hôpital St. Louis." For diseases of the skin, Gibert, St. Louis, in winter, or in the spring the clinical lectures given by the different physicians of St. Louis.

For the general student, the following embraces a good order for visiting the chief hospitals, and attending to the most important subjects :—

*Mondays.* Dr. Gibert—Hôpital St. Louis—Skin diseases and examination with speculum, 8 o'clock.

*Tuesdays.* Dr. P. Dubois—Hôpital des Cliniques—Obstetrics, at 9 o'clock.

*Wednesdays.* Dr. Denouvilliers—Hôpital St. Louis—Surgery and Uterine Diseases, at 8½ o'clock.

*Thursdays.* Dr. Guersant—Hôpital des Enfants Malades—Surgery applied to the diseases of children, visit at 8 and lecture at 9 o'clock.

*Fridays.* Dr. Trousseau—Hotel Dieu—Practical Medicine, at 7¾ o'clock.

*Saturdays.* Dr. Civiale—Hôpital Necker—Diseases of the Urinary Passages and Stone in the bladder, at 9 o'clock. As this hospital is next door to the Children's Hospital, the medical clinique, from 8 to 9, may also be attended at that institution, if the student wishes it.

The usual daily routine of life in Paris with the student, who is really one in deed as well as in name, is to rise before daylight and visit a hospital; then get his breakfast; next take his lesson in French; then perhaps dissect, or attend a lecture at the School of Medicine, and fill up the remainder of the time not allotted to sleep, with reading of French medical books. There are many idlers here, I am sorry to say, who manage to attend one of the cliniques which allow of a little longer sleep in the mornings than those I have referred to, and who thus get a little hospital experience to boast of when they go home, but who waste much of their time in frolicking and sight-seeing, instead of study. These, as a general rule, are either men who are not obliged to depend upon their profession for a living, or those who have very recently obtained their diploma and have as yet had no practical experience in the medical profession. Paris is a bad place to send such men to learn wisdom. They would profit much more by a visit made later in life.

Much might be said about the comparative merits of our own and of the French hospitals, but I leave this to the good sense of the medical stranger, who will no doubt form a correct opinion with regard to the matter. We have, in America, many improvements, particularly in the treatment of fractures and the dressing of wounds, of which the French seem to be entirely ignorant. Their want of language is one great cause of this, together with a rather contemptuous opinion which they hold in regard to our merits as surgeons and physicians. The chief great merit of the French, is their accuracy of diagnosis and the great attention paid to imparting a knowledge of it to others.

Some idea of the extent of the hospitals of Paris may be formed, when I state, that from 80,000 to 100,000 patients are treated in them yearly; that between 4,000 and 5,000 dead subjects are sent from them to the dissecting amphitheatres; and that $2,500,000 are annually expended in their support.

Whilst I am upon this subject of hospitals, I will also state a few facts upon the study of medicine in Europe in general. The best Obstetric clinique, is that of the great General Hospital of Vienna. In this hospital, there are about 8,000 births in the obstetric department per annum, or about 20 births a day on an average throughout the year. The student takes, with 23 others, a course of practical instruction, lasting eight weeks, during which time he is in constant attendance, and receives the cases in his turn. He has also the advantage of examining by the touch all the cases that present themselves, whether under his own immediate care, or that of others. The second Obstetric school of Europe, is the Dublin Lying-in Hospital, where there are 2,000 births per annum. The best ear clinique, is that of Mr. Wilde, of Dublin. At St. Mark's Hospital, there is also a Dispensary for out patients, both eye and ear, under the care of the same surgeon. Mr. Wilde, is no doubt, the best aurist in the world, and he is, perhaps, second to no one in his knowledge of the diseases of the eye. I have attended his clinique, and can speak from experience, with regard to the value of the instruction given. For the diseases of the eye and ear, I would recommend him above any one with whose reputation I am acquainted in Europe.—*Medical Examiner.*

